# In Vivo Evaluation of the Nitroimidazole-Based Thioflavin-T Derivatives as Cerebral Ischemia Markers

**DOI:** 10.1155/2007/49791

**Published:** 2007-08-30

**Authors:** Taiwei Chu, Zejun Li, Xinqi Liu, Xiangyun Wang

**Affiliations:** Bejing National Laboratory for Molecular Sciences (BNLMS), Department of Applied Chemistry, College of Chemistry and Molecular Engineering, Peking University, Beijing 100871, China

## Abstract

Timely imaging and accurate interpretation of cerebral ischemia are required to identify patients who might benefit from more aggressive therapy, and nuclear medicine offers a noninvasive method for demonstrating cerebral ischemia. Three nitroimidazole-based thioflavin-T derivatives, *N*-[4-(benzothiazol-2-yl)phenyl]-3-(4-nitroimidazole-1-yl) propanamide (4NPBTA), *N*-[4-(benzothiazol-2-yl)phenyl]-3-(4-nitroimidazole-1-yl)-*N*-methylpropanamide (4NPBTA-1), and *N*-[4-(benzothiazol-2-yl)phenyl]-3-(2-nitroimidazole-1-yl) propanamide (2NPBTA), were radioiodinated and evaluated as possible cerebral ischemia markers. In normal mice, these compounds showed good permeation of the intact blood-brain barrier (BBB), high initial brain uptake, and rapid washout. In gerbil stroke models that had been subjected to right common carotid artery ligation to produce cerebral ischemia, [^131^I]2NPBTA, uptake in the right cerebral hemisphere decreased more slowly than that of the left, and the right/left hemisphere uptake ratios increased with time. Also, the right/left hemisphere uptake ratios correlated positively with the severity of the stroke. The results showed that [^131^I]2NPBTA had a specific location in the cerebral ischemic tissue. This represented a first step in finding new drugs and might provide a possible cerebral ischemic marker.

## 1. INTRODUCTION

Stroke is the third cause of mortality and the first cause of disability in adults [1, 2]. As cerebral ischemia cannot be predicted, timely imaging and accurate interpretation are required to identify patients who might benefit from more aggressive therapy. Although computed tomography (CT) and magnetic resonance imaging (MRI) have been important and widely used clinical imaging techniques, there are some shortcomings in the imaging of acute stroke, such as the limited brain coverage. Nuclear medicine offers a noninvasive method for demonstrating cerebral ischemia. However, up to now, the markers of cerebral ischemia were scarce [2, 3].

**Figure 1:**
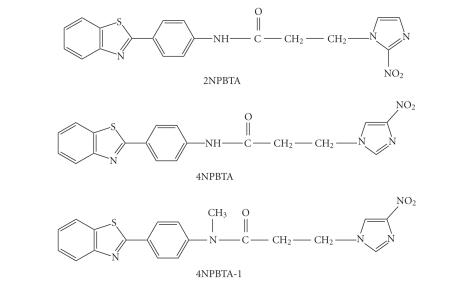
Structures of [^131^I]2NPBTA, [^131^I]4NPBTA, and [^131^I]4NPBTA-1.

The ideal cerebral ischemia markers should not only per-meate across the BBB but also accumulate in the brain ischemia. Recently, Mathis et al. synthesized a series of thio-flavin-T derivatives, which had the “benzothiazole-aniline” backbone and showed good permeation across the BBB [4–6]. Also it is well known that the nitroimidazole derivatives can selectively accumulate in hypoxic tissue and be used to image tumor hypoxia [7–9] and cerebral ischemia [10, 11]. In our previous study, three nitroimidazole-based thioflavin-T derivatives were synthesized and radiolabeled with iodine-131 (see Figure 1): *N* -[4-(benzothiazol-2-yl)-phenyl]-3-(2-nitroimidazole-1-yl) propanamide (2NPBTA), *N*-[4-(benzothiazol-2-yl)phenyl]-3-(4-nitroimidazole-1-yl)-propanamide (4NPBTA), and *N*-[4-(benzothiazol-2-yl) phe-nyl]-3-(4-nitroimidazole-1-yl)-*N*-methylpropanamide (4N-PBTA-1). In vitro and in vivo results showed that they could bind to viable hypoxic tumor cells [12]. In this paper, their permeability across the BBB into the normal brain and *in vivo* evaluation in the gerbil cerebral ischemia models were investigated.

## 2. MATERIALS AND METHODS

No-carrier-added Na[^131^I] (aqueous solution) was obtained from China Institute of Atomic Energy. 2NPBTA, 4NPBTA, and 4NPBTA-1 were synthesized and radiolabeled with iodine-131 in our laboratory [12].

Kunming mice were obtained from Breeding Center of the Institute of Zoology and adult Mongolian gerbils from Breeding Center of Capital University of Medical Sciences. All experiments were carried out following the principles of laboratory animal care and the China law on the protection of animals. Radioactivity in the brain of the animals was assayed using a Cobra II series auto-gamma counting system (Packard).

### 2.1. Brain uptake and clearance in normal mice

Brain uptake and clearance were performed using normal Kunming mice (males, ∼ 20 g). Each mouse received a 0.1 mL (2 *μ*Ci, MBq) dose of [^131^I]2NPBTA, [^131^]4NPBTA, or [^131^I]4NPBTA-1 by tail vein injection. Such injection solution (0.1 mL) was taken as standard for calculating the percent injected dose per gram of tissue, that is, %ID/g. At 2 and 30 minutes postinjection, mice were killed without anesthesia by cervical dislocation in groups of five. The brain was removed, weighed, and counted. The brain uptake (the percent injected dose per gram of tissue, %ID/g) was calculated. The 2-to-30 minutes ratios of %ID/g of the brain were calculated. The final results were expressed as mean ± standard deviation (SD).

### 2.2. Evaluation in gerbil cerebral ischemia models

Adult Mongolian gerbils (males, ∼ 100 g) were used for cerebral ischemia models. A right common carotid artery ligation was performed to produce cerebral hypoxia-ischemia (HI) as initially described by Levine and Payan [13]. Gerbils were anesthetized intraperitoneally (i.p.) with 3.5% chloral hydrate (1 mL), and the four limbs were fixed. A midline anterior incision was made, the right common carotid artery was isolated and ligated with 6–0 surgical sutures distally and proximally. Then the vessel was transected to assure no flow. Thereafter, the incision was closed, and the gerbils were allowed to awake. Gerbils were ranked by the modified stroke index (SI) described by Ohno et al. [14]. Gerbils with total stroke indices of > 10 were used for injection. [^131^I]2NPBTA, [^131^I]4NPBTA, or [^131^I]4NPBTA-1 (0.5 mL, 10 *μ*Ci, MBq) was injected i.p. into the gerbils. Such injection solution (0.5 mL) was taken as standard for calculating the percent injected dose per gram of tissue, that is, %ID/g. Then, animals were housed in controled animal facilities. They were given food and water *ad libitum* . The gerbils were sacrificed without anesthesia by cervical dislocation in groups of three at 4, 8, and 12 hours after injection. The whole brain was removed, placed on dry ice for about 2 minutes, and then cut in half along the cerebral longitudinal fissure. The right and left halves were weighed and radioactivity counted. The %ID/g was determined for the right and left hemispheres. The right/left hemispheral uptake ratios, that is, the ratios of ischemic brain to normal brain, were also calculated. The final results were expressed as mean ± standard deviation (SD). The observed significance level (*P* value) was determined using Student's t-test.

### 2.3. Evaluation of 2NPBTA following repetitive administration

[^131^]2NPBTA (0.1 mL, 2 *μ*Ci) was injected intraperitoneally (i.p.) into the gerbils subjected to right common carotid artery ligation at 0, 60, 120, 180, and 240 minutes. Such injection solution (0.1 mL) was taken as standard for calculating the percent injected dose per gram of tissue, that is, %ID/g. Before sacrifice, the stroke index was determined. Gerbils were sacrificed (no anesthesia) by cervical dislocation 120 minutes after the final injection. The whole brain was removed, blotted free of excess blood, placed on dry ice for about 2 minutes, and then sliced coronally at approximately 3-mm intervals, yielding a total of five coronal slices, designated A–E, from rostral to caudal ends. Each slice was then cut in half at the midsagittal plane, and the right and left halves were weighed and radioactivity counted. The %ID/g of all brain slices was determined.

## 3. RESULTS

### 3.1. Normal brain uptake and clearance

High brain uptake and low nonspecific binding, as measured by the ratio of brain uptake at 2 and 30 minutes, will generally improve the quality of brain tomographic studies [5]. It can be seen from Table 1 that the brain uptake of the three compounds was high at 2 minutes, and low binding at 30 minutes. As a result, the ratio of brain uptake at 2 and 30 minutes for [^131^I]2NPBTA, [^131^I]4NPBTA, and [^131^I]4NPBTA-1 reached 6.2, 9.7, and 5.4, correspondingly. Thus, these compounds showed not only good permeation across the BBB into the brain at the early stage postinjection but also fast clearance from the normal brain tissue soon, making them worthy of further study as brain ischemia markers.

### 3.2. Uptake in ischemic and normal brain hemisphere of gerbil

The uptake of [^131^I]2NPBTA, [^131^I]4NPBTA, and [^131^I]4N-PBTA-1 in the ischemic brain hemisphere (right) and normal brain hemisphere (left) at 4, 8, and 12 hours after administration is presented in Table 2. The right/left (ischemic/normal) hemispheral uptake ratios were also calculated. The uptake in the right hemisphere was higher than that in the left at 4, 8, and 12 hours postinjection for [^131^I]2NPBTA. This indicated that the clearance from ischemic brain tissue was slower than that from normal brain tissue. This was attributed to the selective accumulation of 2-nitroimidazole in hypoxic or ischemic conditions [15, 16]. The right/left uptake ratios, that is, the uptake ratios of ischemic to normal brain tissues were gradually increasing for [^131^I]2NPBTA, from 1.18 at 4 hours to 1.76 at 12 hours. Similar to the finding of Read et al. that 30% of penumbral tissue of ischemic stroke was present at an average of approximately 20 hours using the hypoxia marker ^18^F-labeled fluoromisonidazole [17], the time for [^131^I]2NPBTA was over 12 hours or even longer. As for [^131^I]2NPBTA, the difference between the uptake of the right hemisphere and the left hemisphere was significant (.05 > *P* > .01) at 12 hours, and the difference of the right/left uptake ratios between 12 hours and 4 hours was very significant (*P* < .01).

Unfortunately, as for [^131^I]4NPBTA and [^131^I]4NPBTA-1, the differences between ischemic and normal brain tissues were not significant among all groups (*P* > .05).

### Uptake of [^131^I]2NPBTA following repetitive administration

**Figure 2:**
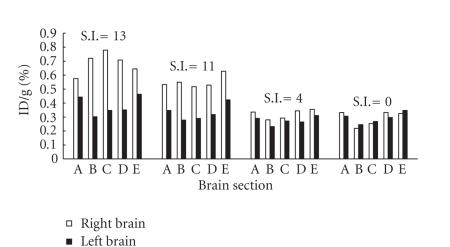
The uptake of [^131^I]2NPBTA in coronal brain slices (designated A, B, C, D, E from rostral to caudal ends) from 4 gerbils subjected to right common carotid ligation after repetitive administration. The coronal slices stroke index (SI) was indicated above the histograms.

Figure 2 shows the uptake of [^131^I]2NPBTA in coronal brain slices from 4 gerbils subjected to right common carotid ligation after multiple injections. Uptake was measured 2 hours after the last injection. The brain of each gerbil was divided into five 3-mm coronal sections, designated A–E, from rostral to caudal ends. The stroke indices ranging from 0 to 13 were indicated above the histograms for each animal. Increased uptake of [^131^I]2NPBTA in ischemic hemispheres was found. The right/left hemisphere uptake ratios were 1.1, 0.9, 0.9, 1.1, and 0.9 from A to E for stroke index = 0 and 1.2, 1.2, 1.1, 1.3, 1.1 for stroke index = 4. For stroke indices = 11 and 13, they were 1.5, 2.0, 1.8, 1.7, 1.5 and 1.3, 2.4, 2.2, 2.0, 1.4. In the gerbil with no evidence of ischemia (stroke index 0), there was no evident right/left difference in any coronal sections. In the gerbil with a stroke index of 4, there was slight right/left difference. The gerbils with markedly symptomatic ischemia (stroke indices 11 or 13) showed a 2-fold or 2.4-fold higher uptake in the midparietal region of the right hemisphere compared with the left. This confirmed that there was greater uptake of [^131^I]2NPBTA in the right hemisphere compared with the left and verified that there was a trend for increased uptake with increasing stroke index. Our data supported the result reported by Hoffman et al. through the study of [^3^ H]misonidazole [10] that the right/left hemisphere uptake ratios correlated positively with the severity of the stroke.

For the gerbils with stroke index 11 or 13, the right/left hemisphere uptake ratios were modest at the most anterior coronal sections, that is, section A, the rostral end. The unique anterior circulation of gerbil cerebral vessels may explain this. The two anterior cerebral arteries of the gerbils fuse in the interhemispheric fissure to form a single pericallosal artery [18–20]. Ischemia may be developed in the anterior brain regions on the nonligated side following the loss of the contributing anterior cerebral flow from the opposite internal carotid circulation [10]. Thus, the rostral end of the left brain of the most symptomatic gerbil also showed an increase in uptake, compared with the corresponding coronal section in gerbil with very low stroke index. The gerbil has an incomplete circle of Willis and, after carotid ligation, may develop severe ischemia in the forebrain. The cerebellum and brain stem, which are supplied by the vertebral arteries, are included in the posterior sections of the brain. They are not ischemic after carotid ligation. Thus, the right/left difference was also modest at the caudal end, that is, the section E, and the coronal brain sections consistently showed a decreasing rostal-to-caudal binding of [^131^I]2NPBTA, from B to E. Different from the result of Hoffman et al. [10] that there was no difference between right and left at the caudal ends, ours also showed difference at slice E, they were 1.5 fold and 1.4 fold for stroke indices = 11 and 13.

## 4. DISCUSSION

The unique anatomical feature of the gerbil made them widely used as a model in global ischemia [18, 19, 21]. Unlike rats, gerbils do not have a posterior communicating artery, that is, the circle of Willis is incomplete and there is incomplete anastomosis of the anterior cerebral arteries. Therefore, the blood supply of each hemisphere is isolated from the contralateral carotid and basilar arteries. Thus, global cerebral ischemia in gerbils can be induced by bilateral common carotid artery occlusion or unilateral common carotid occlusion. Ligation of one carotid artery causes ischemia in the ipsilateral hemisphere, while the other side is unaffected, providing neighboring normal tissue as an internal control. Many investigators have demonstrated that unilateral carotid occulation produces homolateral ischemia and/or infarction in approximately 30–50% of adult male gerbils [18–20].

This study indicated that [^131^I]2NPBTA, [^131^I]4NPBTA, and [^131^I]4NPBTA-1 showed good permeation across the BBB into the brain and fast washout from the normal brain tissue. This study also demonstrated a specific location of [^131^I]2NPBTA in the cerebral ischemic tissue. These results represented a first step toward cerebral ischemia markers of 2NPBTA and made it worthy of further investigation.

## Figures and Tables

**Table 1:** Brain uptake and clearance.

Compound	2 min (ID%/g)	30 min (ID%/g)	Ratio of 2-to-30 min
[^131^I]2NPBTA	2.93 ± 0.39	0.47 ± 0.11	6.2
[^131^I]4NPBTA	2.90 ± 0.31	0.30 ± 0.10	9.7
[^131^I]4NPBTA-1	3.31 ± 0.50	0.61 ± 0.16	5.4

**Table 2:** Uptake in the gerbil ischemic and normal brain hemisphere (%ID/g).

Brain	[^131^I]2NPBTA			[^131^I]4NPBTA			[^131^I]4NPBTA-1		
	4 h	8 h	12 h	4 h	8 h	12 h	4 h	8 h	12 h
Right	0.042 ± 0.005	0.034 ± 0.006	0.025 ± 0.004*	0.046 ± 0.006	0.022 ± 0.001	0.020 ± 0.004	0.040 ± 0.008	0.017 ± 0.001	0.015 ± 0.002
Left	0.036 ± 0.004	0.025 ± 0.003	0.014 ± 0.002	0.038 ± 0.001	0.018 ± 0.005	0.016 ± 0.003	0.034 ± 0.010	0.013 ± 0.002	0.012 ± 0.002
Right/Left	1.18 ± 0.13	1.39 ± 0.10(**)	1.76 ± 0.10(***)	1.22 ± 0.16	1.28 ± 0.28(**)	1.24 ± 0.03(*)	1.18 ± 0.02	1.26 ± 0.28(**)	1.27 ± 0.13(**)

Each value is mean ± SD

(*).05>*P* value >.01 as compared with left brain.

(**)*P* value >.05 as compared with 4 hours.

(***)*P* value <.01 as compared with 4 hours.
